# A Modified Limiting Dilution Method for Monoclonal Stable Cell Line Selection Using a Real-Time Fluorescence Imaging System: A Practical Workflow and Advanced Applications

**DOI:** 10.3390/mps4010016

**Published:** 2021-02-20

**Authors:** Mingyu Ye, Martina Wilhelm, Ivaylo Gentschev, Aladár Szalay

**Affiliations:** 1 Department of Biochemistry and Cancer Therapy Research Center (CTRC), Theodor-Boveri-Institute, University of Wuerzburg, 97074 Wuerzburg, Germany; mingyu.ye@uni-wuerzburg.de (M.Y.); Wilhelm_M3@ukw.de (M.W.); ivaylo.gentschev@mail.uni-wuerzburg.de (I.G.); 2 Department of Radiation Oncology, Rebecca & John Moores Comprehensive Cancer Center, University of California, San Diego, CA 92093, USA

**Keywords:** monoclonal stable cell, limiting dilution cloning, IncuCyte^®^S3

## Abstract

Stable cell lines are widely used in laboratory research and pharmaceutical industry. They are mainly applied in recombinant protein and antibody productions, gene function studies, drug screens, toxicity assessments, and for cancer therapy investigation. There are two types of cell lines, polyclonal and monoclonal origin, that differ regarding their homogeneity and heterogeneity. Generating a high-quality stable cell line, which can grow continuously and carry a stable genetic modification without alteration is very important for most studies, because polyclonal cell lines of multicellular origin can be highly variable and unstable and lead to inconclusive experimental results. The most commonly used technologies of single cell originate monoclonal stable cell isolation in laboratory are fluorescence-activated cell sorting (FACS) sorting and limiting dilution cloning. Here, we describe a modified limiting dilution method of monoclonal stable cell line selection using the real-time fluorescence imaging system IncuCyte^®^S3.

## 1. Introduction

Single cell isolation is a crucial step of many applications, such as monoclonal stable cell line establishment, monoclonal antibodies production, gene editing, and stem cell and chimeric antigen receptor (CAR) T cell therapy. Fluorescence-activated cell sorting (FACS) is a specialized type of flow cytometry used to detect and measure individual cell characteristics based on size, granularity, and fluorescence from a heterogeneous fluid cell mixture [1]. In addition, FACS is one of the most utilized methods for live cell separation [2]. Cells are hydrodynamically focused into a single cell stream of sheath fluid and detected in the core stream when they pass through a focused laser beam [3]. Meanwhile, the cells of interest are selected based on light scattering or emission properties, then deflected into a 96-well plate [4].

Limiting dilution cloning is one of the most common convenient and low-cost methods of single cell isolation [5]. This technique is based on the Poisson distribution, which allows obtaining individual cells quickly and easily from diluted cell suspensions using hand-pipettes or pipetting robots [6,7]. In order to get a sufficient number of individual cells, cell suspension get highly diluted to obtain a density of less than one cell per aliquot (0.5 to 0.9 cell per well in a 96-well plate is the optimal density) [8,9]. To this end, transferring 100 µL of the 5 cells/mL solution into each well of a 96-well plate is the most common model. Unfortunately, using this method can still lead to some wells having multiple cells, which can cause an insufficient number of monoclones from single cells. Moreover, there is no guarantee that the colonies originate from single cells because it is difficult to identify all the clones using a standard microscope. Therefore, limiting dilution cloning normally should be performed at least twice to get a monoclonal cell cluster [10].

IncuCyte^®^S3 is a live-cell imaging system developed by Bioscience, it provides instant and real-time cell distribution images, which help to derive physiologically relevant information about the cells, such as cell confluence or migration. Based on these parameters, real-time kinetic data can be obtained.

## 2. Experimental Design

Enhanced green fluorescence protein (eGFP)-labeled lentivirus infection of N2C cells (primary mammary carcinoma cell line) was followed by blasticidin antibiotic selection. After calculating the density, cells were subsequently diluted to a concentration of 5 cells/mL in suspension and transferred 100 µL per well into six 96-well plates where each well was filled with 100 µL 10% Fetal bovine serum (FBS) DMEM medium. The plates were analyzed after 12 h and 10–14 days by using the whole-well imaging program of IncuCyte^®^S3 to confirm the single cell originate clones. Wells with monoclonal cells only were grown to 80–100% confluency and then transferred to a 24-well plate for amplification. Wells with separated polyclones were identified under the microscope (Zeiss Axiovert 40 CFL Inverted Phase Contrast Microscope) and the undesired clones and cells were eliminated by removing them with a 1–200 µL pipette tip. Wells containing mixed polyclonal cells were seeded into a new 6-well plate with limited dilution and cells were amplified for further 4–7 days followed by marking the clone of interest under the microscopy and eliminating the uninteresting clones and cells. All negative or uninteresting clones and cells were detached using cell scraper and petite tips followed by washing with Phosphate Buffered Saline (PBS), the remaining interesting clones in wells were kept in culture for amplification. Finally, all the monoclonal stable cell lines were seeded into a 24-well plate to establish the cell growth curve and measure the eGFP fluorescence intensity by IncuCyte^®^S3 using the living cell image program.

### 2.1. Materials

- HEK 293T cell line (DSMZ no.: ACC 635) and N2C cell line (kindly provided by Professor Mario P. Colombo, Fondazione IRCCS Istituto Nazionale dei Tumori, Milano, Lombardia, Italy).
- Dulbecco’s Modified Eagle’s Medium-high glucose (Thermo Fisher Scientific, Waltham, MA, USA, 11965092),Opti-MEM™ I Reduced Serum Medium (Thermo Fisher Scientific, 31985062), Fetal Bovine Serum (Merck, Darmstadt, Germany, F4135), Penicillin-Streptomycin (Thermo Fisher Scientific, 15070063), TurboFectin8.0 (Origene, Rockville, MD, USA, TF81001),Hexadimethrine bromide (Merck, Darmstadt, Germany, H9268-5G), Blasticidin solution (InvivoGen, San Diego, CA, USA, ant-bl-05).
- Ultracel-100 regenerated cellulose membrane 15 mL sample volume (Merck, Darmstadt, Germany, UFC910024), Nunc™ 50 mL conical sterile polypropylene centrifuge tubes (Thermo Fisher Scientific, 339652), safe-lock protein oBind tubes (Eppendorf, Hamburg, Germany, 925000090), 96-well plate (Corning, New York, NY, USA, 3596), Corning® 1–200 μL universal fit pipette tips (Merck, Darmstadt, Germany, CLS4860-960EA), cell scrapers (Celltreat, Pepperell, MA, USA, 229310), laboratory markers (Edding, Ahrensburg, Germany, 8015 F 0.75 m Black).
- pTet-turboFP635-EF-1a-eGFP-PKG-BSD plasmid (this study) was constructed from pTet-IRES-eGFP plasmid (kindly provided by Professor Maria Li Lung, Department of Clinical Oncology, Li Ka Shing Faculty of Medicine, the University of Hong Kong (Hong Kong, China). First, turboFP635 gene was inserted into the pTet-IRES-eGFP plasmid at the Pme I restriction site. Second, Human elongation factor-1 alpha (EF-1a) promoter gene was inserted into the plasmid at the Pst I and BmgBI restriction sites, and then the PKG promoter-Blasticidin fusion gene was inserted at the SalI restriction site. tTA_BFP(#58854) plasmid was purchased from Addgene (Watertown, MA, USA).

### 2.2. Equipment

- Incucyte^®^S3 Live-Cell Analysis System (Essen BioScience, Royston, UK; Cat. no.: 4647).
- Zeiss Axiovert 40 CFL inverted phase contrast microscope (Zeiss, Oberkochen, Germany, Suspended).

## 3. Procedure and Results

### 3.1. Lentivirus Production and N2C Cell Infection

#### 3.1.1. Lentivirus Production

##### Cultivation of HEK 293T Cells

One or two days before transformation, plate 2 × 10^6^ HEK293T cells in a 10 cm dish in 10 mL of DMEM supplemented with 10% heat-inactivated fetal bovine serums (FBS) and 1% penicillin-streptomycin and let the cells grow until the reach 70–80% confluence.

##### Prepare DNA/Lentivirus Mixture

**Figure 1:**
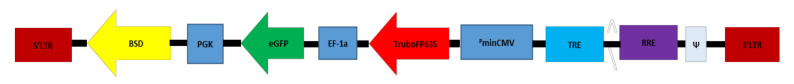
Schematic presentation of lentivirus transfer plasmid pTet-turboFP635-EF-1a-eGFP-PKG-BSD.

In a sterile Eppendorf tube, mix 10 µg pTet-turboFP635-EF-1a-eGFP-PKG-BSD plasmid (Schematic presentation, Figure 1; Plasmid map, Appendix A) and 5 μg (0.5 μg/μL) packaging vectors (PMD2.G and psPAX2) in 1.2 mL of Opti-MEM I medium. Add 40 μL of turbofectin 8.0 (Origene) to the mixture and mix by pipetting. Incubate the mixture for 30 min at room temperature.

##### Transfection of HEK 293T Cells

Add the mixture prepared in step 3.1.1.2 dropwise to the HEK 293T cells and gently rock the plate back and forth and from side to side to distribute the complex evenly. Incubate the cells in a CO_2_ incubator at 37 °C overnight (12 h). Replace the overnight culture medium with fresh DMEM medium supplemented with 2–5% heat-inactivated FBS and 1% penicillin-streptomycin. Collect the virus-containing medium at 36, 48, and 60 h post virus-transfection into a Falcon 50 mL conical centrifuge tube and keep at 4 °C.

##### Harvest of Lentivirus

Centrifuge the falcon tubes at 500× *g* for 10 min to remove the cell debris. Pass the collected supernatants through a 0.45 µM filter and concentrate the virus using Amicon Filter at 3000 rpm for 10–20 min at 4 °C. Store the lentivirus stokes at −80° C.

#### 3.1.2. Lentivirus Infection of N2C Cells

**Figure 2:**
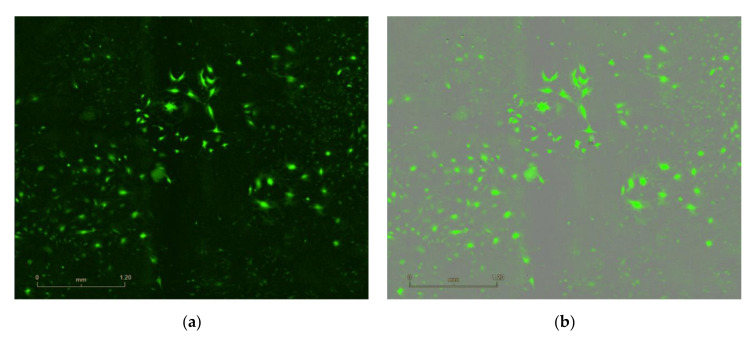
eGFP lentivirus infected N2C cells at seven days post infection. (**a**) Expression of eGFP in infected cells detected by fluorescence with Incucyte^®^S3. (**b**) Overlap of fluorescence image with bright field image. Scale bars represent 1.2 mm.

N2C cells was cultured with lentivirus contained DMEM complete +10 μg/mL polybrene medium, after 72 h incubation, the media was replaced with 10 µg/mL blasticidin (InvivoGen) containing DMEM medium for another 1 week (Figure 2).

### 3.2. Monoclonal Cell Selection after Lentivirus Post-Infection

#### 3.2.1. Seeding of Individual Cells in 96 Well Plates

1. Prepare 60 mL of a 5 cell/mL cell suspensions, then transfer 100 µL of that cell solution into each well of six 96-well plates and incubate at 37 °C in 5% CO_2_ for 10–14 days.

#### 3.2.2. Selection of Monoclonal Cells

**Figure 3:**
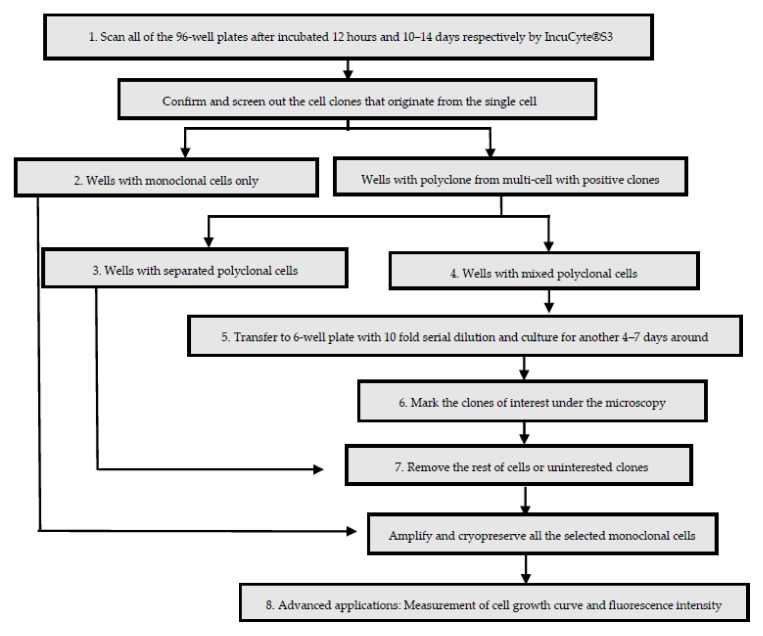
Workflow of monoclonal cells selection after lentivirus infection.

The steps of monoclonal cells selection are showed in the Workflow (Figure 3). The Different Steps in Workflow Marked with Numbers (1–8) in the Rectangles Are Matched with the Followed Procedures (1–8).

**Figure 4:**
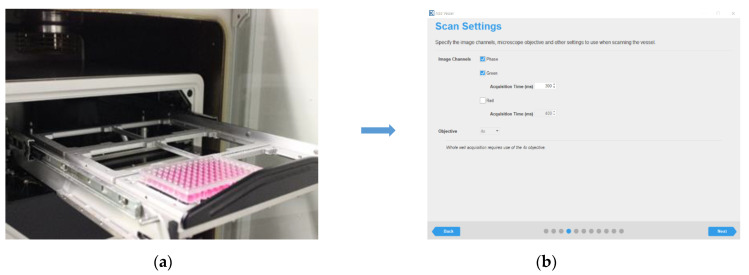

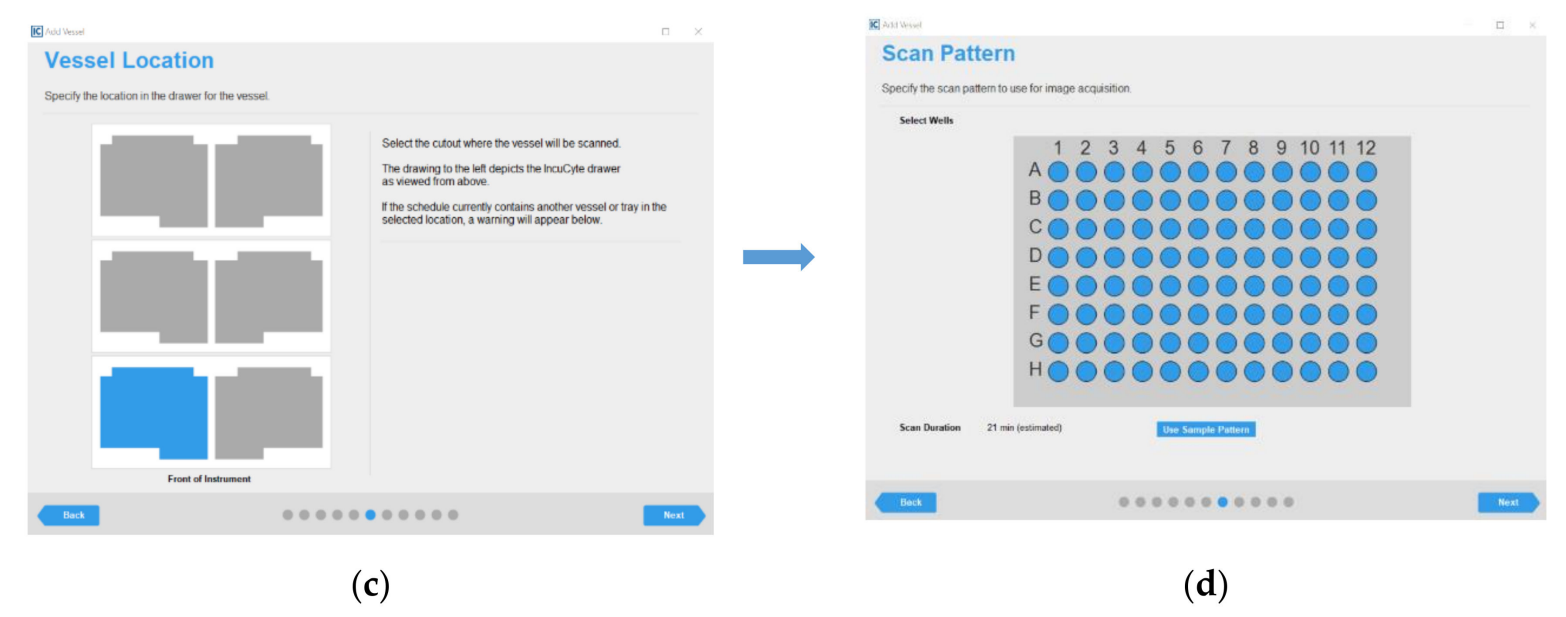
Steps of IncuCyte^®^S3 whole well image for the 96 wells plate scanning. (**a**) Put the 96-well plate into the IncuCyte^®^S3. (**b**) Select the image channels. (**c**) Chose the vessel location. (**d**) Select the wells for scanning.

**Figure 5:**
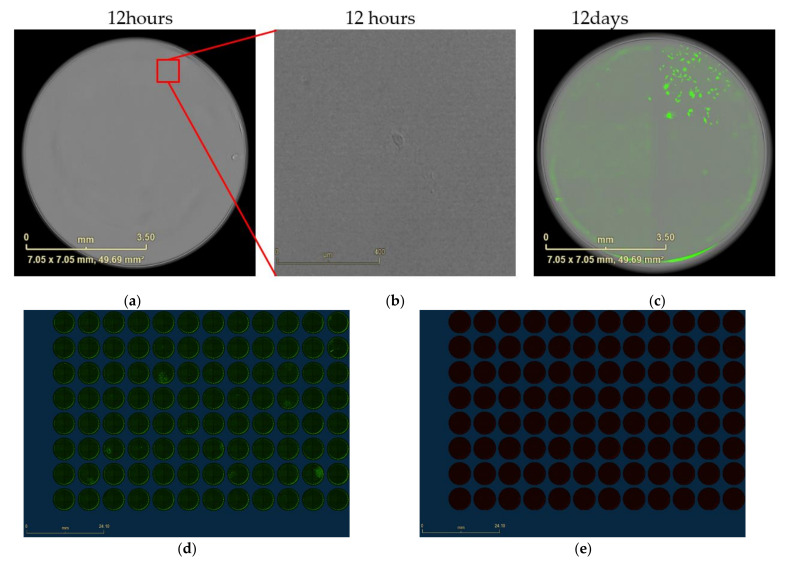
IncuCyte^®^S3 whole well image of the 96-well plate scanning (**a**–**e**). (**a**) Image from 96-well plate scanned after 12 h incubation by bright phase, scale bars represent 3.5 mm. (**b**) Image enlargement (IE) from (**a**), scale bar = 0.4 mm (**c**) The same well scanned after 12 days incubation with overlap of fluorescence image and bright field image. Scale bars represent 3.5 mm. (**d**) Expression of eGFP in diluted cells detected by direct green fluorescence after 12 days incubation. (**e**) Expression of TurboFP635 in diluted cells detected by direct red fluorescence after 12 days incubation. Scale bars represent 24.1 mm.

**Figure 6:**
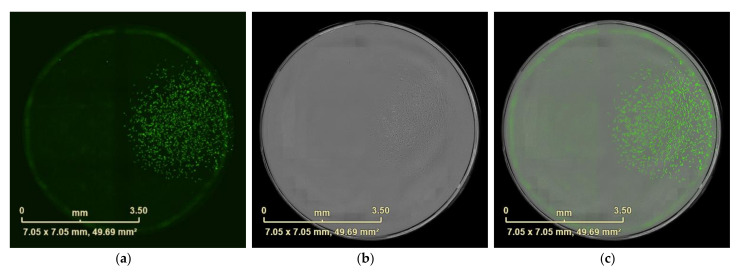
Well with one monoclonal cell only. Image from 96-well plate scanned after 12 days incubation. (**a**) Expression of GFP in cells detected by eGFP fluorescence. (**b**) Bright field images (**c**) Overlap of fluorescence image with bright field image. Scale bars represent 3.5 mm.

**Figure 7:**
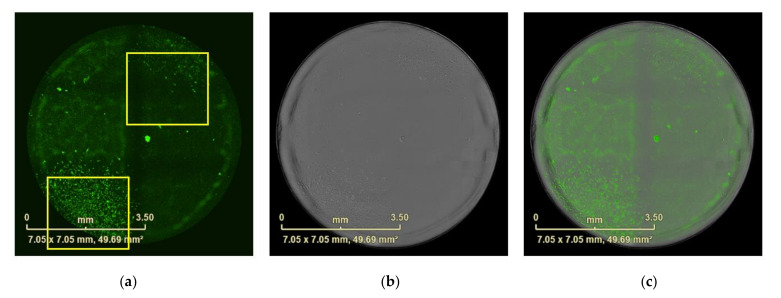
Wells with separated monoclonal cells imaged after 12 days incubation (**a**–**c**). (**a**) Well scanned by eGFP fluorescence. (**b**) Bright field image. (**c**) Overlap of fluorescence image with bright field image. The yellow squares show two separated monoclonal cells, all scale bars represent 3.5 mm.

**Figure 8:**
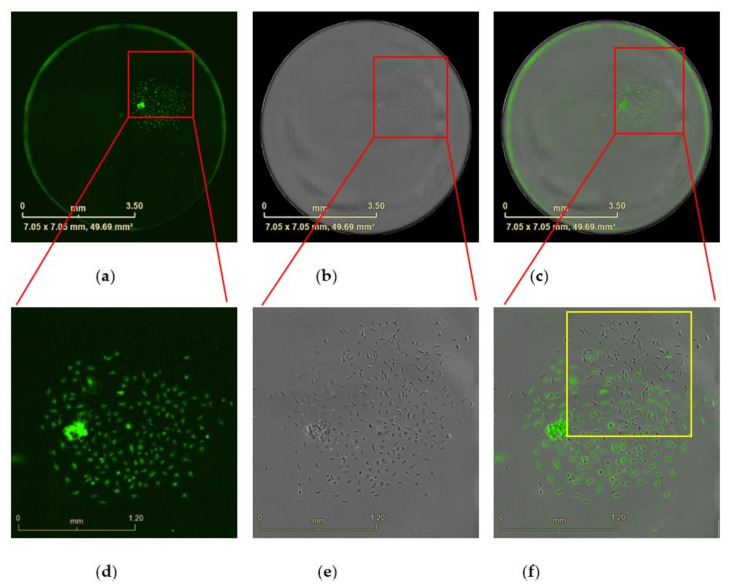
Wells with polyclonal cells in close proximity or mixed with each other. Images from 96-well plate scanned after incubated for 12 days. (**a**) Well scanned by eGFP fluorescence. (**b**) Bright field image. (**c**) Overlap of fluorescence image with bright field image. (**a**–**c**) Scale bars represent 3.5 mm. (**d**) Image enlargement (IE) from (**a**). (**e**) IE from (**b**). (**f**) IE from (**c**). (**d**–**f**) Scale bar = 1.2 mm. The yellow square shows polyclonal cells mix with each other.

**Figure 9:**
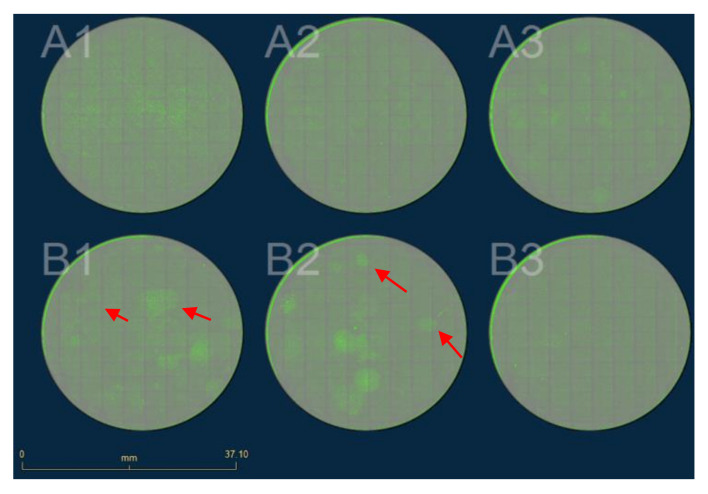
eGFP fluorescence image of polyclonal cells after transfer into 6-well plate with serial dilution. The polyclonal cells are marked by a red arrow. Scale bar = 3.71 cm. Different polyclonal cells are marked by the red arrow.

**Figure 10:**
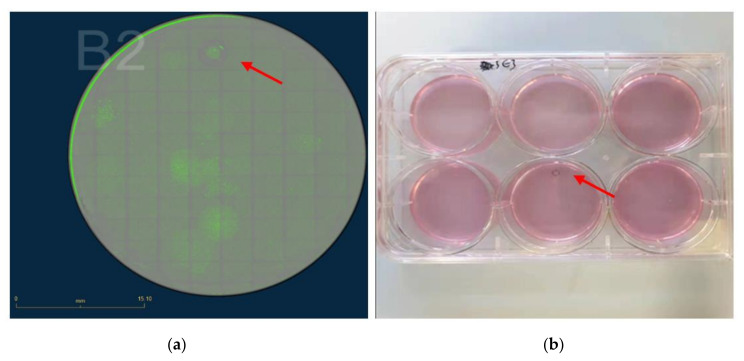
Image of marked monoclonal cells in 6-well plate. (**a**) Well B2 scanned by eGFP fluorescence, Scale bar = 15.1 mm. (**b**) Picture of 6-well plate with marked monoclonal cells. The interesting clone is marked by a red arrow.

**Figure 11:**
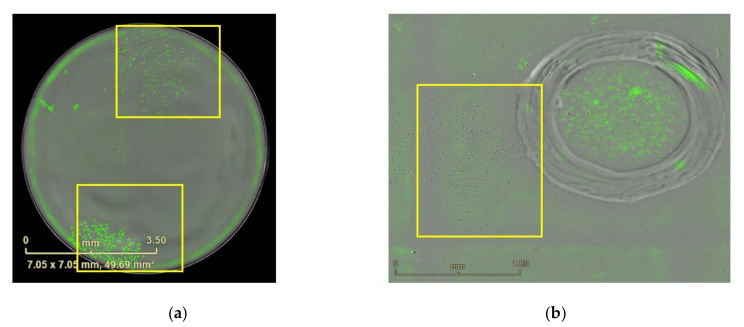
Imaging of the polyclonal cells before elimination. (**a**) Polyclone from 96-well plate. Scale bars = 3.5 mm. The yellow squares show monoclonal cells separated with each other. (**b**) Polyclone from 6-well plate. Scale bars represent 1.8 mm.

**Figure 12:**
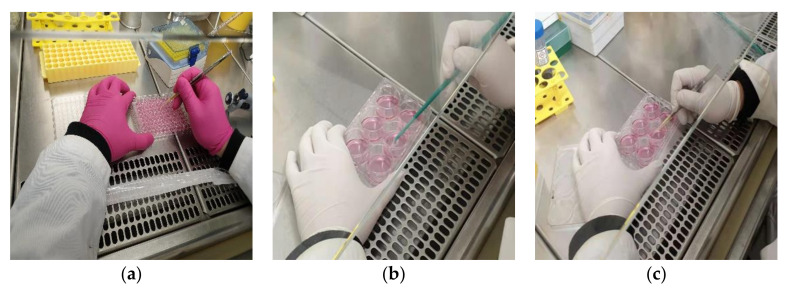
Uninteresting cells eliminate with tips and scraper. Use cell scraper to eliminate the cells at distant location from the marked clone. After that, eliminate the cells that closely surround the marked clone and on the edge of the well carefully by using the pipette tips. (**a**) Cell elimination by using tips in 96-well plate well. (**b**) Cell elimination by using cell scraper in 6-well plate well. (**c**) Cell elimination by using tips in 6-well plate well.

**Figure 13:**
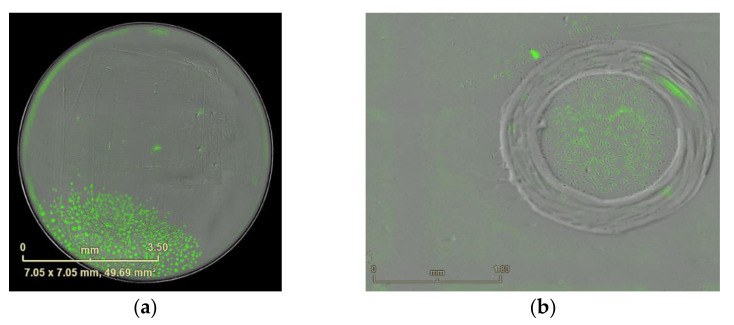
Imaging of the marked clones after elimination. (**a**) Image from Figure 11a after elimination and two days incubation, Scale bar = 3.6 mm. (**b**) Image from Figure 11b after elimination and two days incubation. Scale bar = 1.8 mm.

**Figure 14:**
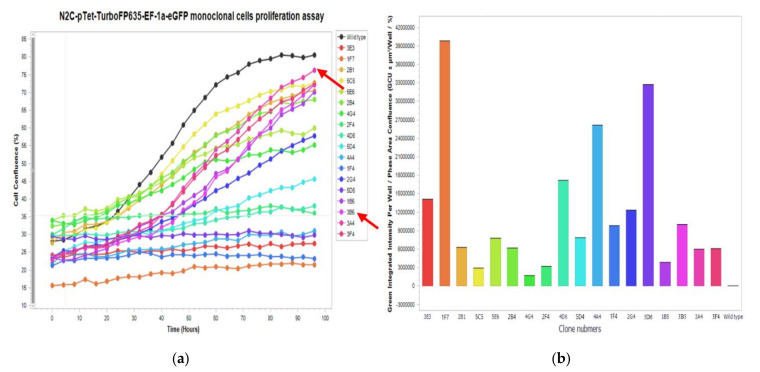
Time-dependent cell growth and eGFP fluorescence intensity of different N2C-pTet-TurboFP635-EF-1a-eGFP monoclonal cells. (**a**) Time-dependent cell growth of different N2C-pTet-TurboFP635-EF-1a-eGFP monoclonal cells. The clone 3A4 is marked by red arrow. (**b**) eGFP fluorescence intensity of different N2C-pTet-TurboFP635-EF-1a-eGFP monoclonal cells.

**Figure 15:**
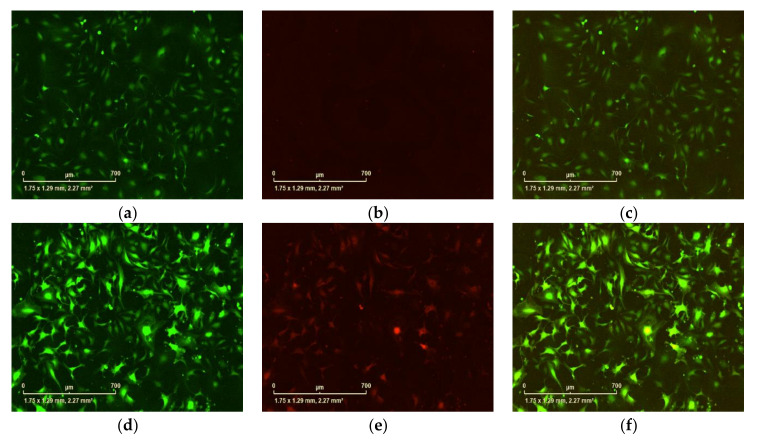
Validation of Tet-off TurboFP635 expression System. N2C-pTet-TurboFP635-EF-1a-eGFP 3A4 cells without transfect with tTA_BFP plasmid ((**a**–**c**), scale bar = 0.7 mm). (**a**) eGFP detection, (**b**) TurboFP635 detection, (**c**) Overlap of green and red fluorescence image; N2C-pTet-Turbo-eGFP 3A4 cells transfect with tTA_BFP plasmid ((**d**–**f**), scale bar = 0.7 mm). (**d**) eGFP detection, (**e**) TurboFP635 detection, (**f**) overlap of green and red fluorescence image.

1. Imaging of 96-well plates (Steps of IncuCyte^®^S3 scanning, Figure 4). The plates were scanned by Incucyte^®^S3 after around 12 h and 10–14 days incubation (Figure 5), then confirm and screen out the cell clones that originate from the single cell.
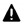
**CRITICAL STEP**. It is important to get the first image after around 12 h incubation, to confirm whether the further cell clones originate from the single cell or not. If the size of cell clones is not big enough after 10–14 days (dependent on the cell type), please prolong the incubation time.
2. Processing of wells have monoclonal cells originate from single cell only (Figure 6). Keep the wells that have only one monoclone for amplifying.
3. Processing of wells have separated monoclonal cells (Figure 7). Go directly to step 7 for eliminating the uninteresting cells.
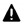
**CRITICAL STEP**. Here we keep the clone (with stronger eGFP expression and bigger size of cell cluster) at the bottom of the image which will be shown in step 7. If you want to keep both clones, use tips to detach one of the clones and transfer the cells with medium to a new 6 well plate.
4. Processing of wells with mixed polyclonal cells in 96-well plate (Figure 8). Go to step 5 (Figure 9): Transfer the cells to a 6-well plate with 10-fold serial dilution and culture for another 4–7 days.
5. Transfer of polyclonal cells from 96-well plate to 6-well plate (Figure 9). Add 150 µL DMEM medium with 10% FBS, after treating the former step (step 4, Figure 3) cells with 50 µL trypsin for 5 min, and transfer the liquid into a 1.5 mL Eppendorf tube with 800 µL medium, wash the well with medium from the tube three times, then transfer the cells into a 6-well plate with 10-fold serial dilution from A1 well to B3 well, make the final volume 2 mL medium per well. Culture the cells another 4–7 days (dependent on cell types).
6. Marking of interesting monoclonal cells (Figure 10). Scan the 6-well plate with the whole well imaging program of the Incucyte^®^S3 and estimate the position of the interesting monoclonal cells according to the image. Then, manually mark the clones in the 6-well plate under the microscopy with marker pen.
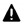
**CRITICAL STEP**. Using a black color mark pen for marking the fluorescence labeled cells. It is not necessary to mark the uninteresting clones in the 96-well plate for cell elimination as the right position can be easily identified.
7. Elimination of uninteresting cells (Figure 11, Figure 12 and Figure 13). For 96-well plates, use pipette tips to detach the cells directly. For 6-well plates, use cell scraper to detach the cells in center of the well and use the 1–200 μL pipette tips to detach the cells on the edge of the well (Figure 12), then wash 3 times with PBS and check under the microscope whether all uninteresting cells were removed (Figure 13).
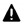
**CRITICAL STEP**. For cell clone elimination, use tips to scratch the uninteresting clones, then wash the well several times with PBS and check the well under the microscope to ensure that all uninteresting cells are eliminated. Caution: Some cells from the interesting clones could be washed away by PBS as well.
8. Advanced applications: Measurement of cell growth curve and fluorescence intensity. Analysis of the isolated monoclonal cells by measurements of cell confluence at different time points and analysis of eGFP fluorescence intensity. Seed 2–4 × 10^4^ cells per well of different monoclonal cells in 24-well plates. Put the plate in the IncuCyte^®^S3 and set up the program for detection of the phase object confluence (Proliferation Assays for Live-Cell Analysis) and eGFP fluorescence intensity (Figure 14). In addition, using this machine, we also validated the tetracycline (Tet)-dependent TurboFP635 expression of one of the optimal monoclonal cells (3A4) by transfect with tTA_BFP plasmid (Figure 15).
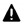
**CRITICAL STEP**. Seed the same numbers of cells in each well. All isolated monoclonal cell lines were tested in confluence and fluorescence assays.

## 4. Discussion

In cloning by limiting dilution, the individual cell can be obtained from in a highly diluted sample through serial dilution due to the statistical distribution of the cells in the suspension. To obtain a sufficiently high probability for single cells while minimizing the probability of the multiple cells, the samples should be diluted to a certain number of cells per aliquot, according to the Poisson’s distribution [11]. However, this method usually results in insufficient monoclonal clone selection and a single cell origin cannot undoubtedly be ensured. Therefore, we believe it is necessary to improve this method. In this paper, we described a modified method for isolation of single cell originated monoclonal stable cell lines base on limiting dilution cloning combined with the IncuCyte^®^S3 system. The IncuCyte^®^S3 is a multi-well plate and flask cell image system enables real-time or instant visualization and quantification of cell characteristics by automatically gathering and analyzing images. In this work, we mainly used the IncuCyte^®^S3 for identification of single cells and clonal populations. Moreover, this system could measure the cell growth curve and fluorescence intensity of different monoclonal cells. This fact enables the identification of optimal clones for further experiments. Our modified method is based on limiting dilution cloning, so it keeps all the advantages the original method has, like stability, reproducibility, ease of manipulation, and ability get high viability clones. Compared to the traditional limiting dilution way, our modified method could ensure that all of the selected cell colonies are originated from the single cells and could rescue some interesting positive monoclonal cells from the polyclones. Although FACS sorting is a popular way to screen single cells separation in research, there are several limiting disadvantages. First, cells collected in this way normally have lower viability and tend to become more easily contaminated due to the rapid flow in the machine and the non-specific fluorescent detection. Second, FACS requires a large number of cells for starting and it is relatively time consuming for the huge amount of cells to be sorted and analyzed [12,13]. In addition, using FACS for single cell isolation is an expensive method and needs a highly trained specialist. Compared to the FACS sorting, our method allows isolation of single cells with lower risk of contaminations, and higher viability (higher population of living cells). The advantages and disadvantages compare with these methods are shown in Table 1. In summary, our study shows that the modified method of limiting dilution cloning combined with the IncuCyte^®^S3 system in this paper can be used as an effective approach for the selection of fluorescence marked and single cell originated clonal cells.

## 5. Conclusions

In this study, we described a modified limiting dilution method of monoclonal stable cell line purification with the help of the IncuCyte^®^S3 system. The procedure can rescue most of positive single clones from the wells and ensure that the analyzed clones originate from individual cell. The method here used the generation of lentiviral green fluorescence protein (eGFP) expression and tetracycline (Tet)-dependent TurboFP635 expression system, and selection of polyclonal cell lines followed by isolation of monoclonal cell lines by means of limiting dilution cloning combined with the IncuCyte^®^S3 imaging system. Using this method, we successfully selected 18 single-cell originate monoclonal cells from six 96-well plates, five of them rescued from the polyclones from the mammary carcinoma N2C cell line (Table 2). We also present advanced applications of the IncuCyte^®^S3 system, such as cell growth curve generation and fluorescence intensity detection, which allows further characterization of single cells. Using these functions, it is possible to pick out the optimal clone based on cell viability together with the fluorescence intensity. Further, we demonstrate the robustness and efficacy of this method by the selection of 18 monoclonal stable cells expressing eGFP in N2C carcinoma cells. Finally, we screened out the best cell clone (designated as 3A4) based on the detected viability and the eGFP fluorescence intensity and validated its tetracycline (Tet)-dependent TurboFP635 expression. These monoclonal cells may be, for instance, useful for further animal tumor model establishment experiments.

## Figures and Tables

**Table 1:** Comparison of modified and traditional limiting dilution cloning and FACS sorting.

	Modified Limiting Dilution Cloning with IncuCyte^®^S3	Traditional Limiting Dilution Cloning	FACS Sorting
**Time-Consuming**	One run	Multiple runs (At least two)	One run
**Labor-Intensity**	Median	High	Low
**Costly Equipment**	Median	Low	High
**Cost consumption**	Median	Low	High
**Cell parameters**	Yes (Fluorescence intensity and cell proliferation rate)	No	Yes (Fluorescence intensity)
**Operability**	Easy	Easy	Hard

**Table 2:** Different monoclonal N2C-pTet-TurboFP635-EF-1a-eGFP cell lines.

Clone Numbers	Type of Clone
1B5	Rescued from polyclonal
1F4	Rescued from polyclonal
1F7	Original monoclonal
2B1	Original monoclonal
2B4	Original monoclonal
2F4	Original monoclonal
2G4	Original monoclonal
3A4	Rescued from polyclonal
3B5	Original monoclonal
3E3	Rescued from polyclonal
3F4	Original monoclonal
4A4	Original monoclonal
4D6	Original monoclonal
4G4	Original monoclonal
5C5	Original monoclonal
5D4	Original monoclonal
5D6	Rescued from polyclonal
5E6	Original monoclonal

**Figure A1:**
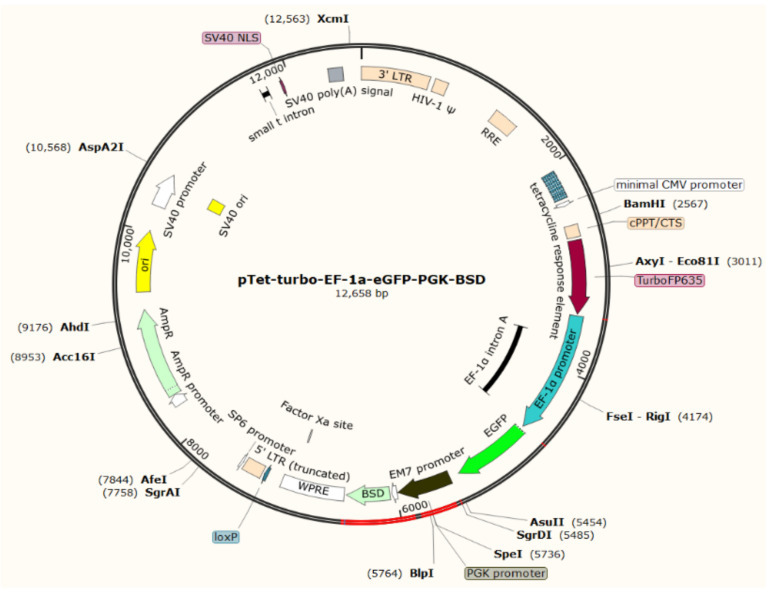
Map of plasmid pTet-turboFP635-EF-1a-eGFP-PKG-BSD.

## Data Availability

The data presented in this study are available on request from the first author.
